# (2*E*,25*E*)-11,14,17,33,36,39,42-Hepta­oxa­penta­cyclo­[41.4.0.0^5,10^.0^18,23^.0^27,32^]hepta­tetra­conta-1(43),2,5(10),6,8,18,20,22,25,27,29,31,44,46-tetra­decaene-4,24-dione

**DOI:** 10.1107/S1600536811013201

**Published:** 2011-04-13

**Authors:** Le Tuan Anh, Truong Hong Hieu, Anatoly T. Soldatenkov, Svetlana A. Soldatova, Victor N. Khrustalev

**Affiliations:** a Department of Chemistry, Vietnam National University, 144 Xuan Thuy, Cau Giay, Hanoi, Vietnam; b Organic Chemistry Department, Russian Peoples Friendship University, Miklukho-Maklaya St 6, Moscow, 117198, Russian Federation; c X-Ray Structural Centre, A. N. Nesmeyanov Institute of Organoelement Compounds, Russian Academy of Sciences, 28 Vavilov St, Moscow 119991, Russian Federation

## Abstract

The title compound, C_40_H_40_O_9_, is a product of the double crotonic condensation of bis­(2-acetyl­phen­oxy)-3-oxapentane with bis­(2-formyl­phen­oxy)-3,6-dioxaoctane. The title macromolecule includes the 31-crown-7-ether skeletal unit and adopts a saddle-like conformation. The two ethyl­ene fragments have *E* configurations. The volume of the inter­nal cavity of the macrocycle is approximately 125 Å^3^. In the crystal, the mol­ecules are arranged at van der Waals distances.

## Related literature

For general background to the design, synthesis and applications of macrocyclic ligands for coordination and supra­molecular chemistry, see: Hiraoka (1978 ▶); Pedersen (1988 ▶); Bradshaw & Izatt (1997) ▶; Gokel & Murillo (1996 ▶). For related compounds, see: Levov *et al.* (2006 ▶, 2008 ▶); Anh *et al.* (2008 ▶)
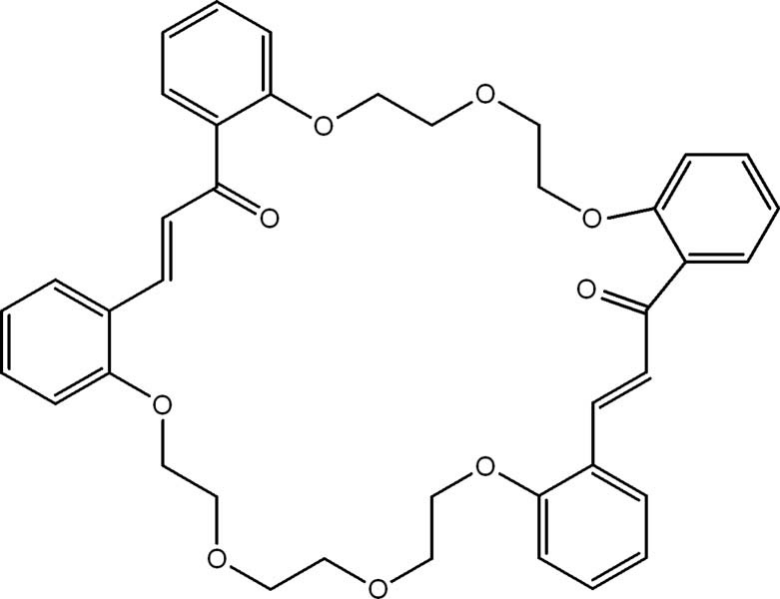


## Experimental

### 

#### Crystal data

C_40_H_40_O_9_
*M* *_r_* = 664.72
Monoclinic, 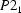

*a* = 12.3268 (6) Å
*b* = 11.0271 (6) Å
*c* = 13.1142 (7) Å
β = 106.933 (1)°
*V* = 1705.32 (15) Å^3^
*Z* = 2
Mo *K*α radiation
μ = 0.09 mm^−1^
*T* = 120 K
0.30 × 0.30 × 0.20 mm

#### Data collection

Bruker SMART 1K CCD diffractometer
Absorption correction: multi-scan (*SADABS*; Sheldrick, 1998 ▶) *T*_min_ = 0.973, *T*_max_ = 0.982
19455 measured reflections
5222 independent reflections
4511 reflections with *I* > 2σ(*I*)
*R*_int_ = 0.027

#### Refinement

*R*[*F* ^2^ > 2σ(*F* ^2^)] = 0.051
*wR*(*F* ^2^) = 0.128
*S* = 1.01
5222 reflections
442 parameters
1 restraint
H-atom parameters constrained
Δρ_max_ = 0.33 e Å^−3^
Δρ_min_ = −0.20 e Å^−3^

### 

Data collection: *SMART* (Bruker, 1998 ▶); cell refinement: *SAINT-Plus* (Bruker, 1998 ▶); data reduction: *SAINT-Plus*; program(s) used to solve structure: *SHELXTL* (Sheldrick, 2008 ▶); program(s) used to refine structure: *SHELXTL*; molecular graphics: *SHELXTL*; software used to prepare material for publication: *SHELXTL*.

## Supplementary Material

Crystal structure: contains datablocks global, I. DOI: 10.1107/S1600536811013201/rk2273sup1.cif

Structure factors: contains datablocks I. DOI: 10.1107/S1600536811013201/rk2273Isup2.hkl

Additional supplementary materials:  crystallographic information; 3D view; checkCIF report

## Figures and Tables

**Fig. 1.**
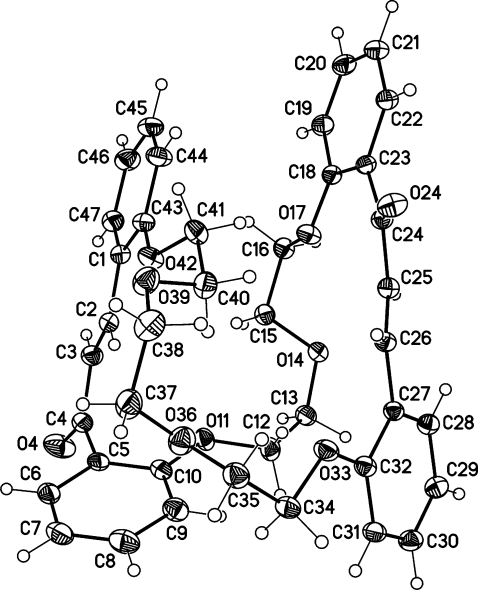
Molecular structure of I with the atom numbering scheme. Displacement ellipsoids are shown at the 50% probability level. H atoms are presented as a small spheres of arbitrary radius.

**Fig. 2.**
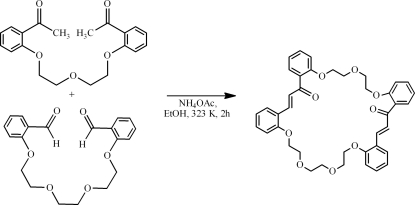
Domino cyclocondensation of bis(2-acetylphenoxy)-3-oxapentane with bis(2-formylphenoxy)-3,6-dioxaoctane.

## supplementary crystallographic information

### Comment

Design, synthesis and applications of macrocyclic ligands for coordination and supramolecular chemistry draw very great attention of investigators during the last forty years (Hiraoka, 1978; Pedersen, 1988; Gokel & Murillo, 1996; Bradshaw & Izatt, 1997). Recently, we have developed an effective method of synthesis of 14- and 17-membered azacrown (Levov *et al.*, 2006; 2008) and crown (Anh *et al.*, 2008) ethers. This method is based on domino reaction of three components - dialkyl ketone, bis(2-formylphenoxy)-3-oxapentane and ammonium acetate, *i.e.*, the modified Petrenko–Kritchenko reaction (Levov, 2008).

In attempts to apply this chemistry for obtaining of a ditopic ligand, in which two azacrown units are connected to each other by polyether chain, we studied the similar condensation of bis(2-formylphenoxy)-3,6-dioxaoctane with bis(2-acetylphenoxy)-3-oxapentane and ammonium acetate, the latter being both a source of nitrogen and a template agent. However, instead of the expected azacrown system, tetrakis(benzo)-31-crown-7-ether (I) was formed.

The obtained compound I, C_40_H_40_O_9_, includes the 31–crown–7–ether skeletal moiety and adopts a saddle-like conformation (Fig. 1). The two ethylene fragments have *E*configurations. The dihedral angles between the benzene planes of C1,C43–C47/C5–C10, C5–C10/C18–C23, C18–C23/C27–C32 and C27–C32/C1,C43–C47 are 64.91 (8), 65.14 (8), 61.64 (8) and 56.67 (9)°, respectively. The volume of the internal cavity of macrocycle I is approximately equal to 125 Å^3^. The distances from the center of macrocycle cavity, defined as centroid of O11/O14/O17/O33/O36/O39/O42 oxygen donor atoms, to the O11, O14, O17, O33, O36, O39 and O42 oxygen atoms are 3.286 (3), 3.638 (3), 3.460 (3), 3.308 (3), 3.486 (3), 3.524 (3) and 2.533 (3) Å, respectively.

In the crystal, the molecules of I are arranged at van der Waals distances.

### Experimental

Ammonium acetate (2.0 g, 26 mmol) was added to a solution of bis(2-formylphenoxy)-3,6-dioxaoctane (1.38 g, 4.40 mmol) with bis(2-acetylphenoxy)-3-oxapentane (1.50 g, 4.40 mmol) in ethanol (50 ml). The reaction mixture was stirred at 323 K for 2 h (monitoring by *TLC* until disappearance of the starting organic compounds spots). At the end of the reaction, the formed wax-like precipitate was separated, washed with cold ethanol (50 ml) and re-crystallized from ethanol to give 0.82 g of light-yellow crystals of I (Fig. 2). Yield is 28%. *M*.p. = 400–402 K. IR (KBr), ν/cm^-1^: 1618, 1682. ^1^H NMR (CDCl_3_ , 400 MHz, 300 K): δ = 3.54, 3.62, 3.85 and 4.11 (all m, 6H, 5H, 5H and 4H, respectively, OCH_2_CH_2_O), 6.70–7.23 and 7.28–7.55 (both m, 10H and 6H, respectively, H_arom_), 7.27 and 7.87 (both d, 2H each, O═C—CH*_trans_*═CH, *J* = 16.0). Anal. Calcd for C_40_H_40_O_9_: C, 72.29; H, 6.03. Found: C, 72.31; H, 6.12.

### Refinement

The 4537 Friedel pairs were merged in the refinement procedure. The hydrogen atoms were placed in calculated positions with C—H = 0.95–0.99Å and refined in the riding model with fixed isotropic displacement parameters *U*_iso_(H) = 1.2*U*_eq_(C).

### Crystal data

**Table:**

C_40_H_40_O_9_	*F*(000) = 704
*M**_r_* = 664.72	*D*_x_ = 1.294 Mg m^−^^3^
Monoclinic, *P*2_1_	Melting point = 400–402 K
Hall symbol: P 2yb	Mo *K*α radiation, λ = 0.71073 Å
*a* = 12.3268 (6) Å	Cell parameters from 7007 reflections
*b* = 11.0271 (6) Å	θ = 2.5–29.6°
*c* = 13.1142 (7) Å	µ = 0.09 mm^−^^1^
β = 106.933 (1)°	*T* = 120 K
*V* = 1705.32 (15) Å^3^	Prism, light–yellow
*Z* = 2	0.30 × 0.30 × 0.20 mm

### Data collection

**Table:**

Bruker SMART 1K CCD diffractometer	5222 independent reflections
Radiation source: fine-focus sealed tube	4511 reflections with *I* > 2σ(*I*)
graphite	*R*_int_ = 0.027
φ and ω scans	θ_max_ = 30.0°, θ_min_ = 2.0°
Absorption correction: multi-scan (*SADABS*; Sheldrick, 1998)	*h* = −16→17
*T*_min_ = 0.973, *T*_max_ = 0.982	*k* = −15→15
19455 measured reflections	*l* = −18→18

### Refinement

**Table:**

Refinement on *F*^2^	Primary atom site location: structure-invariant direct methods
Least-squares matrix: full	Secondary atom site location: difference Fourier map
*R*[*F*^2^ > 2σ(*F*^2^)] = 0.051	Hydrogen site location: inferred from neighbouring sites
*wR*(*F*^2^) = 0.128	H-atom parameters constrained
*S* = 1.01	*w* = 1/[σ^2^(*F*_o_^2^) + (0.06*P*)^2^ + 0.86*P*] where *P* = (*F*_o_^2^ + 2*F*_c_^2^)/3
5222 reflections	(Δ/σ)_max_ < 0.001
442 parameters	Δρ_max_ = 0.33 e Å^−^^3^
1 restraint	Δρ_min_ = −0.20 e Å^−^^3^

### Special details

**Table:**

Geometry. All s.u.'s (except the s.u. in the dihedral angle between two l.s. planes) are estimated using the full covariance matrix. The cell s.u.'s are taken into account individually in the estimation of s.u.'s in distances, angles and torsion angles; correlations between s.u.'s in cell parameters are only used when they are defined by crystal symmetry. An approximate (isotropic) treatment of cell s.u.'s is used for estimating s.u.'s involving l.s. planes.
Refinement. Refinement of *F*^2^ against ALL reflections. The weighted *R*–factor w*R* and goodness of fit *S* are based on *F*^2^, conventional *R*–factors *R* are based on *F*, with *F* set to zero for negative *F*^2^. The threshold expression of *F*^2^ > 2σ(*F*^2^) is used only for calculating *R*–factors(gt) *etc*. and is not relevant to the choice of reflections for refinement. *R*–factors based on *F*^2^ are statistically about twice as large as those based on *F*, and *R*–factors based on ALL data will be even larger.

### Fractional atomic coordinates and isotropic or equivalent isotropic displacement parameters (Å^2^)

**Table:**

	*x*	*y*	*z*	*U*_iso_*/*U*_eq_	
C1	0.4588 (2)	0.6322 (2)	0.41234 (19)	0.0280 (5)	
C2	0.3718 (2)	0.6146 (3)	0.46784 (18)	0.0280 (5)	
H2	0.3347	0.5381	0.4603	0.034*	
C3	0.3411 (2)	0.6979 (3)	0.5280 (2)	0.0330 (5)	
H3	0.3786	0.7742	0.5375	0.040*	
C4	0.2520 (2)	0.6771 (3)	0.5802 (2)	0.0325 (5)	
O4	0.2392 (2)	0.7497 (3)	0.64613 (19)	0.0559 (7)	
C5	0.1818 (2)	0.5638 (3)	0.55711 (19)	0.0290 (5)	
C6	0.1917 (2)	0.4805 (3)	0.6388 (2)	0.0359 (6)	
H6	0.2416	0.4975	0.7075	0.043*	
C7	0.1304 (3)	0.3732 (3)	0.6222 (3)	0.0422 (7)	
H7	0.1392	0.3165	0.6786	0.051*	
C8	0.0566 (3)	0.3495 (3)	0.5232 (3)	0.0438 (7)	
H8	0.0149	0.2759	0.5112	0.053*	
C9	0.0426 (3)	0.4326 (3)	0.4402 (2)	0.0371 (6)	
H9	−0.0099	0.4163	0.3726	0.044*	
C10	0.1056 (2)	0.5392 (2)	0.4565 (2)	0.0295 (5)	
O11	0.09816 (16)	0.62631 (18)	0.38125 (13)	0.0306 (4)	
C12	0.0157 (2)	0.6097 (3)	0.2789 (2)	0.0321 (5)	
H12A	−0.0607	0.5992	0.2879	0.039*	
H12B	0.0342	0.5363	0.2438	0.039*	
C13	0.0177 (2)	0.7199 (3)	0.2117 (2)	0.0309 (5)	
H13A	−0.0582	0.7298	0.1598	0.037*	
H13B	0.0322	0.7923	0.2585	0.037*	
O14	0.10022 (15)	0.71629 (19)	0.15489 (13)	0.0313 (4)	
C15	0.2133 (2)	0.7259 (3)	0.22173 (19)	0.0305 (5)	
H15A	0.2341	0.6520	0.2660	0.037*	
H15B	0.2204	0.7965	0.2698	0.037*	
C16	0.2910 (2)	0.7410 (2)	0.15298 (19)	0.0294 (5)	
H16A	0.2647	0.8082	0.1016	0.035*	
H16B	0.3692	0.7589	0.1974	0.035*	
O17	0.28744 (16)	0.62817 (17)	0.09792 (14)	0.0308 (4)	
C18	0.34907 (19)	0.6155 (2)	0.02800 (18)	0.0257 (4)	
C19	0.4309 (2)	0.6979 (3)	0.0181 (2)	0.0314 (5)	
H19	0.4429	0.7709	0.0583	0.038*	
C20	0.4949 (2)	0.6732 (3)	−0.0504 (2)	0.0369 (6)	
H20	0.5507	0.7298	−0.0567	0.044*	
C21	0.4791 (2)	0.5674 (3)	−0.1099 (2)	0.0368 (6)	
H21	0.5234	0.5515	−0.1568	0.044*	
C22	0.3976 (2)	0.4848 (3)	−0.1002 (2)	0.0305 (5)	
H22	0.3881	0.4110	−0.1392	0.037*	
C23	0.32910 (19)	0.5088 (2)	−0.03355 (18)	0.0248 (4)	
C24	0.2399 (2)	0.4172 (2)	−0.03145 (19)	0.0266 (5)	
O24	0.25237 (16)	0.31193 (18)	−0.05580 (17)	0.0365 (4)	
C25	0.1368 (2)	0.4551 (2)	−0.00417 (19)	0.0258 (4)	
H25	0.1164	0.5384	−0.0079	0.031*	
C26	0.07174 (19)	0.3731 (2)	0.02578 (18)	0.0246 (4)	
H26	0.0963	0.2910	0.0314	0.030*	
C27	−0.03407 (19)	0.4002 (2)	0.05046 (18)	0.0238 (4)	
C28	−0.0994 (2)	0.5025 (2)	0.0089 (2)	0.0275 (5)	
H28	−0.0736	0.5571	−0.0351	0.033*	
C29	−0.2012 (2)	0.5259 (2)	0.0307 (2)	0.0310 (5)	
H29	−0.2447	0.5955	0.0016	0.037*	
C30	−0.2387 (2)	0.4466 (3)	0.0954 (2)	0.0328 (5)	
H30	−0.3085	0.4620	0.1100	0.039*	
C31	−0.1751 (2)	0.3444 (3)	0.13935 (19)	0.0298 (5)	
H31	−0.2012	0.2909	0.1839	0.036*	
C32	−0.0731 (2)	0.3219 (2)	0.11732 (18)	0.0263 (5)	
O33	−0.00317 (15)	0.22654 (18)	0.15923 (14)	0.0312 (4)	
C34	−0.0337 (2)	0.1520 (2)	0.2361 (2)	0.0304 (5)	
H34A	−0.1030	0.1049	0.2013	0.036*	
H34B	−0.0489	0.2031	0.2925	0.036*	
C35	0.0637 (2)	0.0675 (3)	0.2836 (2)	0.0346 (5)	
H35A	0.0397	0.0026	0.3246	0.042*	
H35B	0.0897	0.0294	0.2265	0.042*	
O36	0.15233 (17)	0.1363 (2)	0.35162 (16)	0.0397 (5)	
C37	0.2517 (2)	0.0645 (3)	0.3974 (2)	0.0430 (7)	
H37A	0.2280	−0.0189	0.4091	0.052*	
H37B	0.2929	0.0987	0.4679	0.052*	
C38	0.3315 (3)	0.0587 (3)	0.3289 (3)	0.0433 (7)	
H38A	0.3875	−0.0066	0.3561	0.052*	
H38B	0.2873	0.0370	0.2552	0.052*	
O39	0.39042 (17)	0.1687 (2)	0.32615 (17)	0.0398 (5)	
C40	0.3283 (2)	0.2565 (3)	0.2534 (2)	0.0355 (6)	
H40A	0.2639	0.2867	0.2770	0.043*	
H40B	0.2978	0.2202	0.1817	0.043*	
C41	0.4075 (2)	0.3590 (3)	0.2498 (2)	0.0335 (5)	
H41A	0.4825	0.3265	0.2503	0.040*	
H41B	0.3769	0.4066	0.1836	0.040*	
O42	0.41902 (17)	0.43507 (18)	0.34075 (15)	0.0338 (4)	
C43	0.4779 (2)	0.5409 (2)	0.3452 (2)	0.0291 (5)	
C44	0.5554 (2)	0.5598 (3)	0.2867 (2)	0.0375 (6)	
H44	0.5687	0.4978	0.2416	0.045*	
C45	0.6126 (2)	0.6697 (3)	0.2950 (2)	0.0399 (6)	
H45	0.6640	0.6831	0.2544	0.048*	
C46	0.5952 (2)	0.7595 (3)	0.3620 (2)	0.0402 (6)	
H46	0.6354	0.8340	0.3681	0.048*	
C47	0.5192 (2)	0.7412 (3)	0.4201 (2)	0.0339 (5)	
H47	0.5077	0.8035	0.4659	0.041*	

### Atomic displacement parameters (Å^2^)

**Table:**

	*U*^11^	*U*^22^	*U*^33^	*U*^12^	*U*^13^	*U*^23^
C1	0.0239 (10)	0.0335 (13)	0.0271 (10)	−0.0007 (10)	0.0083 (8)	0.0022 (10)
C2	0.0250 (10)	0.0333 (12)	0.0254 (10)	−0.0011 (9)	0.0068 (8)	0.0013 (10)
C3	0.0306 (12)	0.0400 (15)	0.0298 (11)	−0.0059 (11)	0.0110 (9)	−0.0065 (11)
C4	0.0299 (11)	0.0424 (15)	0.0266 (11)	−0.0012 (11)	0.0106 (9)	−0.0046 (11)
O4	0.0670 (15)	0.0603 (16)	0.0529 (13)	−0.0158 (13)	0.0372 (12)	−0.0260 (12)
C5	0.0264 (10)	0.0362 (13)	0.0286 (11)	0.0054 (10)	0.0148 (9)	−0.0008 (10)
C6	0.0312 (12)	0.0477 (16)	0.0329 (12)	0.0137 (12)	0.0156 (10)	0.0084 (12)
C7	0.0428 (15)	0.0457 (17)	0.0458 (16)	0.0144 (13)	0.0252 (13)	0.0179 (13)
C8	0.0499 (17)	0.0360 (15)	0.0527 (17)	−0.0003 (13)	0.0264 (14)	0.0066 (13)
C9	0.0418 (14)	0.0332 (14)	0.0385 (14)	−0.0047 (12)	0.0154 (11)	−0.0005 (11)
C10	0.0324 (11)	0.0319 (12)	0.0275 (11)	0.0007 (10)	0.0137 (9)	0.0015 (10)
O11	0.0372 (9)	0.0307 (9)	0.0228 (7)	−0.0078 (8)	0.0071 (7)	−0.0014 (7)
C12	0.0358 (12)	0.0325 (13)	0.0257 (11)	−0.0056 (10)	0.0051 (9)	−0.0024 (10)
C13	0.0315 (12)	0.0341 (13)	0.0291 (11)	0.0037 (10)	0.0118 (9)	0.0036 (10)
O14	0.0298 (8)	0.0393 (10)	0.0261 (8)	0.0015 (8)	0.0101 (6)	0.0012 (8)
C15	0.0306 (12)	0.0336 (12)	0.0275 (11)	−0.0016 (10)	0.0087 (9)	−0.0029 (10)
C16	0.0322 (12)	0.0271 (12)	0.0291 (11)	−0.0037 (10)	0.0095 (9)	−0.0045 (9)
O17	0.0362 (9)	0.0279 (9)	0.0334 (9)	−0.0060 (8)	0.0184 (7)	−0.0049 (7)
C18	0.0238 (10)	0.0283 (11)	0.0254 (10)	−0.0002 (9)	0.0076 (8)	0.0027 (9)
C19	0.0294 (11)	0.0316 (13)	0.0334 (12)	−0.0070 (10)	0.0096 (9)	0.0020 (10)
C20	0.0267 (11)	0.0442 (15)	0.0403 (13)	−0.0087 (11)	0.0108 (10)	0.0056 (12)
C21	0.0284 (12)	0.0488 (17)	0.0373 (13)	−0.0010 (12)	0.0158 (10)	0.0035 (13)
C22	0.0254 (11)	0.0375 (13)	0.0301 (11)	0.0028 (10)	0.0103 (9)	0.0036 (10)
C23	0.0205 (9)	0.0283 (11)	0.0260 (10)	0.0010 (9)	0.0076 (8)	0.0027 (9)
C24	0.0239 (10)	0.0280 (11)	0.0286 (11)	−0.0001 (9)	0.0087 (8)	0.0029 (9)
O24	0.0329 (9)	0.0272 (9)	0.0522 (12)	−0.0004 (8)	0.0169 (8)	−0.0042 (8)
C25	0.0266 (10)	0.0236 (11)	0.0281 (11)	−0.0014 (9)	0.0093 (8)	−0.0020 (9)
C26	0.0254 (10)	0.0237 (11)	0.0261 (10)	−0.0018 (9)	0.0098 (8)	−0.0005 (8)
C27	0.0244 (10)	0.0241 (11)	0.0240 (10)	−0.0031 (9)	0.0089 (8)	−0.0016 (8)
C28	0.0287 (11)	0.0249 (11)	0.0302 (11)	−0.0022 (9)	0.0105 (9)	−0.0012 (9)
C29	0.0293 (11)	0.0277 (12)	0.0364 (13)	0.0014 (10)	0.0105 (9)	−0.0012 (10)
C30	0.0288 (11)	0.0361 (14)	0.0363 (13)	0.0003 (10)	0.0140 (10)	−0.0044 (11)
C31	0.0295 (11)	0.0331 (13)	0.0300 (11)	−0.0038 (10)	0.0137 (9)	−0.0012 (10)
C32	0.0293 (11)	0.0255 (11)	0.0261 (10)	−0.0036 (9)	0.0111 (9)	−0.0015 (9)
O33	0.0317 (9)	0.0336 (9)	0.0317 (9)	0.0023 (8)	0.0145 (7)	0.0088 (8)
C34	0.0329 (12)	0.0295 (12)	0.0316 (12)	−0.0031 (10)	0.0139 (9)	0.0046 (10)
C35	0.0382 (13)	0.0329 (13)	0.0336 (12)	−0.0050 (11)	0.0118 (10)	0.0026 (11)
O36	0.0379 (10)	0.0381 (11)	0.0405 (10)	−0.0052 (9)	0.0074 (8)	−0.0026 (9)
C37	0.0390 (14)	0.0422 (16)	0.0427 (15)	−0.0061 (13)	0.0042 (12)	0.0122 (13)
C38	0.0405 (15)	0.0318 (14)	0.0543 (17)	−0.0010 (12)	0.0088 (13)	0.0054 (13)
O39	0.0334 (9)	0.0371 (11)	0.0448 (11)	−0.0034 (8)	0.0051 (8)	0.0071 (9)
C40	0.0351 (13)	0.0358 (14)	0.0324 (12)	−0.0018 (11)	0.0048 (10)	0.0008 (11)
C41	0.0403 (13)	0.0349 (13)	0.0270 (11)	−0.0023 (11)	0.0126 (10)	−0.0025 (10)
O42	0.0402 (10)	0.0333 (10)	0.0333 (9)	−0.0066 (8)	0.0190 (8)	−0.0048 (8)
C43	0.0260 (11)	0.0337 (13)	0.0305 (11)	−0.0014 (10)	0.0126 (9)	0.0002 (10)
C44	0.0354 (13)	0.0450 (16)	0.0385 (13)	−0.0044 (12)	0.0208 (11)	−0.0052 (12)
C45	0.0312 (12)	0.0495 (17)	0.0437 (14)	−0.0076 (12)	0.0182 (11)	0.0005 (13)
C46	0.0343 (13)	0.0412 (16)	0.0471 (15)	−0.0120 (12)	0.0149 (12)	−0.0015 (13)
C47	0.0313 (12)	0.0373 (14)	0.0333 (12)	−0.0032 (11)	0.0097 (10)	−0.0012 (11)

### Geometric parameters (Å, °)

**Table:**

C1—C47	1.401 (4)	C24—C25	1.477 (3)
C1—C43	1.402 (4)	C25—C26	1.341 (3)
C1—C2	1.474 (3)	C25—H25	0.9500
C2—C3	1.335 (4)	C26—C27	1.463 (3)
C2—H2	0.9500	C26—H26	0.9500
C3—C4	1.472 (3)	C27—C28	1.402 (3)
C3—H3	0.9500	C27—C32	1.411 (3)
C4—O4	1.222 (3)	C28—C29	1.389 (3)
C4—C5	1.500 (4)	C28—H28	0.9500
C5—C6	1.389 (4)	C29—C30	1.389 (4)
C5—C10	1.405 (3)	C29—H29	0.9500
C6—C7	1.387 (5)	C30—C31	1.397 (4)
C6—H6	0.9500	C30—H30	0.9500
C7—C8	1.376 (5)	C31—C32	1.392 (3)
C7—H7	0.9500	C31—H31	0.9500
C8—C9	1.394 (4)	C32—O33	1.369 (3)
C8—H8	0.9500	O33—C34	1.433 (3)
C9—C10	1.391 (4)	C34—C35	1.504 (4)
C9—H9	0.9500	C34—H34A	0.9900
C10—O11	1.361 (3)	C34—H34B	0.9900
O11—C12	1.440 (3)	C35—O36	1.413 (3)
C12—C13	1.505 (4)	C35—H35A	0.9900
C12—H12A	0.9900	C35—H35B	0.9900
C12—H12B	0.9900	O36—C37	1.435 (4)
C13—O14	1.426 (3)	C37—C38	1.515 (5)
C13—H13A	0.9900	C37—H37A	0.9900
C13—H13B	0.9900	C37—H37B	0.9900
O14—C15	1.418 (3)	C38—O39	1.420 (4)
C15—C16	1.504 (3)	C38—H38A	0.9900
C15—H15A	0.9900	C38—H38B	0.9900
C15—H15B	0.9900	O39—C40	1.417 (3)
C16—O17	1.433 (3)	C40—C41	1.504 (4)
C16—H16A	0.9900	C40—H40A	0.9900
C16—H16B	0.9900	C40—H40B	0.9900
O17—C18	1.358 (3)	C41—O42	1.431 (3)
C18—C19	1.391 (3)	C41—H41A	0.9900
C18—C23	1.407 (3)	C41—H41B	0.9900
C19—C20	1.384 (4)	O42—C43	1.366 (3)
C19—H19	0.9500	C43—C44	1.405 (3)
C20—C21	1.386 (4)	C44—C45	1.390 (4)
C20—H20	0.9500	C44—H44	0.9500
C21—C22	1.388 (4)	C45—C46	1.382 (4)
C21—H21	0.9500	C45—H45	0.9500
C22—C23	1.407 (3)	C46—C47	1.384 (4)
C22—H22	0.9500	C46—H46	0.9500
C23—C24	1.499 (3)	C47—H47	0.9500
C24—O24	1.226 (3)		
			
C47—C1—C43	118.6 (2)	C26—C25—H25	119.7
C47—C1—C2	121.7 (2)	C24—C25—H25	119.7
C43—C1—C2	119.6 (2)	C25—C26—C27	125.1 (2)
C3—C2—C1	124.9 (2)	C25—C26—H26	117.5
C3—C2—H2	117.5	C27—C26—H26	117.5
C1—C2—H2	117.5	C28—C27—C32	118.2 (2)
C2—C3—C4	123.1 (3)	C28—C27—C26	121.8 (2)
C2—C3—H3	118.5	C32—C27—C26	120.0 (2)
C4—C3—H3	118.5	C29—C28—C27	121.3 (2)
O4—C4—C3	119.7 (3)	C29—C28—H28	119.3
O4—C4—C5	120.2 (2)	C27—C28—H28	119.3
C3—C4—C5	120.0 (2)	C30—C29—C28	119.4 (2)
C6—C5—C10	118.7 (3)	C30—C29—H29	120.3
C6—C5—C4	118.6 (2)	C28—C29—H29	120.3
C10—C5—C4	122.6 (2)	C29—C30—C31	120.8 (2)
C7—C6—C5	121.5 (3)	C29—C30—H30	119.6
C7—C6—H6	119.3	C31—C30—H30	119.6
C5—C6—H6	119.3	C32—C31—C30	119.4 (2)
C8—C7—C6	119.3 (3)	C32—C31—H31	120.3
C8—C7—H7	120.3	C30—C31—H31	120.3
C6—C7—H7	120.3	O33—C32—C31	123.7 (2)
C7—C8—C9	120.7 (3)	O33—C32—C27	115.5 (2)
C7—C8—H8	119.7	C31—C32—C27	120.8 (2)
C9—C8—H8	119.7	C32—O33—C34	117.32 (19)
C10—C9—C8	119.9 (3)	O33—C34—C35	107.8 (2)
C10—C9—H9	120.1	O33—C34—H34A	110.2
C8—C9—H9	120.1	C35—C34—H34A	110.2
O11—C10—C9	124.6 (2)	O33—C34—H34B	110.2
O11—C10—C5	115.5 (2)	C35—C34—H34B	110.2
C9—C10—C5	119.9 (2)	H34A—C34—H34B	108.5
C10—O11—C12	117.8 (2)	O36—C35—C34	107.8 (2)
O11—C12—C13	108.4 (2)	O36—C35—H35A	110.1
O11—C12—H12A	110.0	C34—C35—H35A	110.1
C13—C12—H12A	110.0	O36—C35—H35B	110.1
O11—C12—H12B	110.0	C34—C35—H35B	110.1
C13—C12—H12B	110.0	H35A—C35—H35B	108.5
H12A—C12—H12B	108.4	C35—O36—C37	112.1 (2)
O14—C13—C12	114.8 (2)	O36—C37—C38	113.4 (2)
O14—C13—H13A	108.6	O36—C37—H37A	108.9
C12—C13—H13A	108.6	C38—C37—H37A	108.9
O14—C13—H13B	108.6	O36—C37—H37B	108.9
C12—C13—H13B	108.6	C38—C37—H37B	108.9
H13A—C13—H13B	107.6	H37A—C37—H37B	107.7
C15—O14—C13	113.40 (18)	O39—C38—C37	113.9 (3)
O14—C15—C16	108.72 (19)	O39—C38—H38A	108.8
O14—C15—H15A	109.9	C37—C38—H38A	108.8
C16—C15—H15A	109.9	O39—C38—H38B	108.8
O14—C15—H15B	109.9	C37—C38—H38B	108.8
C16—C15—H15B	109.9	H38A—C38—H38B	107.7
H15A—C15—H15B	108.3	C40—O39—C38	114.8 (2)
O17—C16—C15	105.9 (2)	O39—C40—C41	107.8 (2)
O17—C16—H16A	110.6	O39—C40—H40A	110.1
C15—C16—H16A	110.6	C41—C40—H40A	110.1
O17—C16—H16B	110.6	O39—C40—H40B	110.1
C15—C16—H16B	110.6	C41—C40—H40B	110.1
H16A—C16—H16B	108.7	H40A—C40—H40B	108.5
C18—O17—C16	119.23 (19)	O42—C41—C40	108.8 (2)
O17—C18—C19	124.0 (2)	O42—C41—H41A	109.9
O17—C18—C23	115.7 (2)	C40—C41—H41A	109.9
C19—C18—C23	120.2 (2)	O42—C41—H41B	109.9
C20—C19—C18	119.8 (3)	C40—C41—H41B	109.9
C20—C19—H19	120.1	H41A—C41—H41B	108.3
C18—C19—H19	120.1	C43—O42—C41	117.28 (19)
C19—C20—C21	121.2 (3)	O42—C43—C1	117.1 (2)
C19—C20—H20	119.4	O42—C43—C44	122.8 (2)
C21—C20—H20	119.4	C1—C43—C44	120.1 (2)
C20—C21—C22	119.1 (2)	C45—C44—C43	119.8 (3)
C20—C21—H21	120.4	C45—C44—H44	120.1
C22—C21—H21	120.4	C43—C44—H44	120.1
C21—C22—C23	121.0 (3)	C46—C45—C44	120.3 (3)
C21—C22—H22	119.5	C46—C45—H45	119.8
C23—C22—H22	119.5	C44—C45—H45	119.8
C22—C23—C18	118.5 (2)	C45—C46—C47	120.1 (3)
C22—C23—C24	117.5 (2)	C45—C46—H46	120.0
C18—C23—C24	124.0 (2)	C47—C46—H46	120.0
O24—C24—C25	120.9 (2)	C46—C47—C1	121.1 (3)
O24—C24—C23	119.0 (2)	C46—C47—H47	119.5
C25—C24—C23	120.1 (2)	C1—C47—H47	119.5
C26—C25—C24	120.7 (2)		
			
C47—C1—C2—C3	1.0 (4)	C22—C23—C24—C25	−153.5 (2)
C43—C1—C2—C3	−175.4 (3)	C18—C23—C24—C25	27.4 (3)
C1—C2—C3—C4	178.7 (2)	O24—C24—C25—C26	20.1 (4)
C2—C3—C4—O4	169.5 (3)	C23—C24—C25—C26	−162.1 (2)
C2—C3—C4—C5	−7.0 (4)	C24—C25—C26—C27	−177.5 (2)
O4—C4—C5—C6	−62.2 (4)	C25—C26—C27—C28	24.2 (4)
C3—C4—C5—C6	114.2 (3)	C25—C26—C27—C32	−156.2 (2)
O4—C4—C5—C10	117.0 (3)	C32—C27—C28—C29	−1.2 (4)
C3—C4—C5—C10	−66.5 (3)	C26—C27—C28—C29	178.4 (2)
C10—C5—C6—C7	1.7 (4)	C27—C28—C29—C30	0.3 (4)
C4—C5—C6—C7	−179.1 (2)	C28—C29—C30—C31	0.5 (4)
C5—C6—C7—C8	−1.1 (4)	C29—C30—C31—C32	−0.3 (4)
C6—C7—C8—C9	−0.5 (4)	C30—C31—C32—O33	177.9 (2)
C7—C8—C9—C10	1.6 (5)	C30—C31—C32—C27	−0.6 (4)
C8—C9—C10—O11	−179.8 (3)	C28—C27—C32—O33	−177.2 (2)
C8—C9—C10—C5	−1.0 (4)	C26—C27—C32—O33	3.1 (3)
C6—C5—C10—O11	178.3 (2)	C28—C27—C32—C31	1.4 (3)
C4—C5—C10—O11	−0.9 (3)	C26—C27—C32—C31	−178.3 (2)
C6—C5—C10—C9	−0.6 (4)	C31—C32—O33—C34	−4.8 (3)
C4—C5—C10—C9	−179.8 (2)	C27—C32—O33—C34	173.7 (2)
C9—C10—O11—C12	3.0 (4)	C32—O33—C34—C35	−170.8 (2)
C5—C10—O11—C12	−175.9 (2)	O33—C34—C35—O36	72.6 (3)
C10—O11—C12—C13	176.5 (2)	C34—C35—O36—C37	−177.4 (2)
O11—C12—C13—O14	86.1 (3)	C35—O36—C37—C38	88.0 (3)
C12—C13—O14—C15	−69.8 (3)	O36—C37—C38—O39	71.8 (3)
C13—O14—C15—C16	−171.1 (2)	C37—C38—O39—C40	−81.9 (3)
O14—C15—C16—O17	−67.8 (3)	C38—O39—C40—C41	−172.4 (2)
C15—C16—O17—C18	179.1 (2)	O39—C40—C41—O42	−79.6 (3)
C16—O17—C18—C19	11.0 (3)	C40—C41—O42—C43	−172.9 (2)
C16—O17—C18—C23	−171.5 (2)	C41—O42—C43—C1	159.6 (2)
O17—C18—C19—C20	175.6 (2)	C41—O42—C43—C44	−20.9 (4)
C23—C18—C19—C20	−1.7 (4)	C47—C1—C43—O42	179.0 (2)
C18—C19—C20—C21	0.0 (4)	C2—C1—C43—O42	−4.5 (3)
C19—C20—C21—C22	−0.2 (4)	C47—C1—C43—C44	−0.6 (4)
C20—C21—C22—C23	1.9 (4)	C2—C1—C43—C44	175.9 (2)
C21—C22—C23—C18	−3.5 (4)	O42—C43—C44—C45	−179.9 (3)
C21—C22—C23—C24	177.4 (2)	C1—C43—C44—C45	−0.4 (4)
O17—C18—C23—C22	−174.1 (2)	C43—C44—C45—C46	1.2 (5)
C19—C18—C23—C22	3.4 (3)	C44—C45—C46—C47	−1.0 (5)
O17—C18—C23—C24	4.9 (3)	C45—C46—C47—C1	0.0 (4)
C19—C18—C23—C24	−177.6 (2)	C43—C1—C47—C46	0.8 (4)
C22—C23—C24—O24	24.2 (3)	C2—C1—C47—C46	−175.7 (3)
C18—C23—C24—O24	−154.8 (2)		
